# Quinoa Soluble Fiber and Quercetin Alter the Composition of the Gut Microbiome and Improve Brush Border Membrane Morphology In Vivo (*Gallus gallus*)

**DOI:** 10.3390/nu14030448

**Published:** 2022-01-20

**Authors:** Nikita Agarwal, Nikolai Kolba, Noa Khen, Carmel Even, Sondra Turjeman, Omry Koren, Elad Tako

**Affiliations:** 1 Department of Food Science, Cornell University, Ithaca, NY 14850, USA; na494@cornell.edu (N.A.); nk598@cornell.edu (N.K.); nk647@cornell.edu (N.K.); 2 Azrieli Faculty of Medicine, Bar-Ilan University, Safed 1311502, Israel; carmel.even@gmail.com (C.E.); sondra.turjeman@biu.ac.il (S.T.); omry.koren@biu.ac.il (O.K.)

**Keywords:** in vivo, polyphenols, quinoa, microbiome, gene expression, quercetin, mineral deficiency, inulin

## Abstract

Quinoa (*Chenopodium quinoa* Willd.), a gluten-free pseudo-cereal, has gained popularity over the last decade due to its high nutritional value. Quinoa is a rich source of proteins, carbohydrates, fibers, tocopherols (Vitamin E), unsaturated fatty acids and a wide range of polyphenols. The study used *Gallus gallus* intra-amniotic feeding, a clinically validated method, to assess the effects of quinoa soluble fiber (QSF) and quercetin 3-glucoside (Q3G) versus control. Quercetin is a pharmacologically active polyphenol found in quinoa. Six groups (no injection, 18 Ω H_2_O, 5% inulin, 1% Q3G, 5% QSF, 1% Q3G + 5% QSF) were assessed for their effect on the brush border membrane (BBM) functionality, intestinal morphology and cecal bacterial populations. Our results showed a significant (*p* < 0.05) improvement in BBM morphology, particularly goblet and Paneth cell numbers, in the group administered with quinoa and quercetin. However, there were no significant changes seen in the expression of the genes assessed both in the duodenum and liver between any of the treatment groups. Furthermore, fibrous quinoa increased the concentration of probiotic *L. plantarum* populations compared to the control (H_2_O). In conclusion, quercetin and quinoa fiber consumption has the potential to improve intestinal morphology and modulate the microbiome.

## 1. Introduction

Quinoa (*Chenopodium quinoa* Willd.) is a gluten-free pseudo-cereal grown in the Andean region (mainly Peru and Bolivia) of South America. It is referred to as a “pseudo-cereal” as it does not belong to the *Poaceae* family but to the *Chenopodiaceae* family i.e., non-grass. Quinoa provides a range of nutrients from essential amino acids, proteins, lipids, vitamins, minerals, fibers to health-promoting phytocompounds such as alkylresorcinols [1], saponins [2], polyphenols, phytosterols and phytoecdysteriods [3] among others. It is advertised as a “superfood” and promoted as an agricultural crop for its ability to adapt to different soil types and stress-conditions. Being a gluten-free cereal provides an added advantage to quinoa as it is well-tolerated by individuals with gluten intolerance. The United Nations declared 2013 as the “International Year of Quinoa” [4]. Despite these benefits, quinoa is not widely consumed. This may be due to its heavy import prices, lack of awareness among consumers and insufficient research [3,5].

In comparison to rice, wheat, corn, rye and sorghum, quinoa proves to be a better source of protein, lipids, and inorganic minerals with a lower glycemic load [5,6,7]. Whole-grain white quinoa flour comprises of about 14–20% dietary fiber of which soluble fibers range between 3.7% to 5.9% [8]. Arabinans and homogalacturonans are the main soluble fibers reported in quinoa [9]. These fibers can act as prebiotic substances that resist digestion by the host enzymes and undergo fermentation by the gut microbiota [10]. The resident microbes consume the fibers and produce a range of beneficial metabolites including Short-Chain Fatty Acids (SCFAs) [11]. SCFAs such as acetic, butyric, and propionic acid increase intestinal acidity favoring the growth of certain bacterial populations. These SCFAs, when taken up by enterocytes, improve BBM functionality by up-regulating epithelial cell differentiation and increasing villus surface area. Once in the circulating blood, SCFAs can also impact immune function and inflammation in various tissues [12].

In addition to its fiber content, quinoa has a range of beneficial phytocompounds namely quercetin, kaempferol, rutin, myricetin, catechin, coumaric acid, benzoic acid, ascorbic acid, and tocopherol among others [13]. These compounds can influence health directly through their uptake in the intestine or by modifying the intestinal microbiome. Recent studies have concluded that phytocompounds and their metabolites exert a prebiotic effect on beneficial bacteria and an antibacterial effect against the pathogenic bacteria in the gut [14,15,16,17]. Quercetin, one of the main bioactive phytocompound found in quinoa, has been shown to positively affect the gut microbiome in vivo in the context of gut dysbiosis [18], atherosclerosis [19,20], inflammation [21] and obesity [22].

Bioavailability is defined as the portion of the food consumed that can be absorbed by the intestine and made available either for storage or metabolic processes. Zinc and iron have low bioavailability and hence their deficiencies are prevalent, affecting 17% and 40% of the global population respectively [23,24,25]. Merely increasing the consumption of foods rich in the two micronutrients does not guarantee their uptake. Antinutrients such as phytates, found in certain foods, form insoluble complexes with minerals, hindering mineral absorption. Thus, food interventions that improve the brush border membrane (BBM) functionality and alter intestinal microbiota are alternative approaches in improving mineral absorption [26]. People across the world, especially those suffering from malabsorption, heavily rely on cereal-based foods for meeting their daily caloric intake. Regular cereal-based foods including polished wheat, rice and corn are insufficient in fighting iron and zinc nutrient deficiencies [27].

Recent studies have shown that fibers and polyphenols may have a significant effect on immune modulation especially improving gut health [28,29]. However, the mechanisms through which these phytocompounds contribute to health benefits in quinoa seeds are not well established [13]. The objective of this study is to evaluate quinoa soluble fiber (QSF) and quercetin 3-glucoside (Q3G) for their potential prebiotic effect on the microbiome, BBM functionality and mineral absorption, to better understand the mechanisms behind the reported biological effects. Our previous paper, on another gluten-free cereal grain teff (*Eragrostis tef*), demonstrated a significant improvement in iron and zinc status biomarker, up-regulated related genes and brought about beneficial morphometric changes in the intestine including increase goblet cell number and villi surface area [30]. The study was conducted on *Gallus gallus*, a clinically validated intra-amniotic model [22,31,32,33,34,35,36,37]. For the present study, we hypothesize that the intra-amniotic administration of quinoa soluble fiber will have a synergistic effect with quercetin 3-glucoside; favorably altering the intestinal bacterial populations and BBM morphology, and regulating gene function.

## 2. Materials and Methods

### 2.1. Quinoa Soluble Extract Preparation

Washed whole-grain white quinoa seeds were purchased from a grocery store in Ithaca, NY and used in this study. The extraction was performed as previously described [30,37]. Briefly, the quinoa seeds were ground and dissolved in 50 g/L distilled water at 60 °C for 60 min. Then, they were centrifuged at 3000× *g* at 4 °C for 25 min. This removed any particulate matter. The remaining supernatant was exhaustively dialyzed (MWCO 12–14 kDa) for 48 h against distilled water. The dialysate so collected was lyophilized which resulted in an off-white powder.

### 2.2. Animals and Study Design

Cornish-cross fertile broiler eggs (*n* = 60) were obtained from a commercial hatchery (Moyer’s chicks, Quakertown, PA, USA) and incubated until hatch at the Cornell University Animal Science poultry farm. All animal protocols were approved by Cornell University Institutional Animal Care and Use committee (protocol number: 2020-0077). All methods were performed in accordance with the relevant guidelines and regulations. For intra-amniotic administration, 60 eggs containing viable embryos were weighed and allocated to six groups (n~10) randomly. At day 17 of incubation, the injection spot was sanitized, and 1 mL of the soluble water extracts were injected into the amniotic fluid with the help of a 21-gauge needle. The six treatments so given were (1) no injection, (2) 18 Ω H_2_O, (3) 5% inulin, (4) 1% quercetin 3-glucoside, (5) 5% quinoa fiber and (6) 1% quercetin 3-glucoside + 5% quinoa fiber. After injection, the spot was sealed with cellophane tape and the eggs were incubated separately until day 21 (hatch day) [36,38]. The hatchlings were euthanized in a CO_2_ chamber and sample collection was done. The blood, duodenum, liver and cecum were removed and immediately placed in liquid nitrogen (ice for blood) temporarily. The samples were then transferred to a −80 °C incubator until analysis.

### 2.3. Blood Analysis and Hb Measurements

Blood was collected from the heart in micro-hematocrit heparinized capillary tubes. The tubes (Fisher Scientific, Waltham, MA, USA) were shaken to mix the tube’s heparin with the blood collected and placed on ice. Blood Hb concentrations were determined using a spectrophotometer (QuantiChrom™ Hemoglobin Assay DIHB-250, BioAssay Systems, Hayward, CA, USA) the kit manufacturer’s instructions were followed.

### 2.4. Gene Expression Analysis

#### 2.4.1. Isolation of Total RNA from Duodenum and Liver

Total RNA was extracted from 30 mg of duodenum and liver samples (*n* = 6) as described earlier [37,39]. The Qiagen RNeasy Mini Kit (RNeasy Mini Kit, Qiagen Inc., Valencia, CA, USA) was used according to the manufacturer’s protocol. All steps in the protocol were carried out under RNase-free conditions. RNA was quantified by absorbance at A 260/280. Integrity of the 18S ribosomal rRNA was verified by 1.5% agarose gel electrophoresis followed by ethidium bromide staining. DNA contamination was removed using TURBO DNase treatment and removal kit from AMBION (Austin, TX, USA).

#### 2.4.2. Real-Time Polymerase Chain Reaction (RT-PCR)

From the extracted RNA, cDNA was created by a 20 μL reverse transcriptase (RT) reaction. The BioRad C1000 touch thermocycler (BioRad, Hercules, CA, USA), using the Improm-II Reverse Transcriptase Kit (Catalog #A1250; Promega, Madison, WI, USA) was employed. A total of 1 μg of total RNA template with 10 μM of random hexamer primers, and 2 mM of oligo-dT primers were added to the given vial. The annealing step of the primers to RNA occurred at 94 °C for 5 min, copying occurred at 60 min at 42 °C (optimum temperature for the enzyme), then heat inactivation was carried out for 15 min at 70 °C. The cDNA thus obtained was stored at −80 °C until analyses by Nanodrop (Thermo Fisher Scientific, Waltham, MA, USA). cDNA concentration was determined by measuring the absorbance at 260 nm and 280 nm using an extinction coefficient of 33 (for single stranded DNA). Genomic DNA contamination was assessed by a real-time RT-PCR assay for the reference gene samples.

#### 2.4.3. Primer Design

The primers were designed using Real-Time Primer Design Tool software (IDT DNA, Coralvilla, IA, USA) based on 15 gene sequences from the Genebank database. The primer sequences and descriptions can be found summarized in Table 1. The lengths of the primers were 17–25-mer, the GC content was between 41% and 55% and the amplicon length was restricted to 90–150 bp. Primer specificity was verified by BLAST searches against the genomic National Center for Biotechnology Information (NCBI) database. The *Gallus gallus* primer 18S rRNA served as a reference gene.

#### 2.4.4. RT-PCR Design

Performed as previously described [40]. cDNA (2 μL) was pipetted into a 96-well plate with 2× BioRad SSO Advanced Universal SYBR Green Supermix (8 μL) (Cat #1725274, BioRad, Hercules, CA, USA) including buffer, dNTPs, Taq polymerase and dye. Primers (as shown in Table 1) both forward and reserve, cDNA (or water as control) were added to each PCR reaction. Each run had duplicates of 7 standard curve points. A control with no template was included with nuclear-free water to detect and exclude any possible DNA contamination. DNA amplification was done in BioRad CFX96 Touch (BioRad, Hercules, CA, USA) under the following conditions: initial denaturing at 95 °C for 30 s, 40 cycles of denaturing at 95 °C for 15 s, various annealing temperatures according to Integrated DNA Technologies (IDT) for 30 s and elongating at 60 °C for 30 s. The gene expression data were obtained as the lowest cyclic product (Cp) values based on the automated method of “second derivative maximum”. All results were quantified against the standard curve prepared by a 1:10 dilution. For each of the genes, the reactions were run in duplicates. A graph of Cq vs. log (10) concentrations was produced by the software and the efficiencies were calculated as 10 [1/slope]. The specificity of the amplified real-time RT-PCR products was verified by melting curve analysis (60–95 °C) after 40 cycles, resulting in several different specific products, each with a specific melting temperature. Real-time RT-PCR efficiency (E) values for the 15 genes were as follows: DMT-1, 0.998; DcytB, 1.046; Ferroportin, 1.109; 18S rRNA, 0.994, Δ-6 Desaturase, 0.925; ZnT7, 0.916; ZIP6, 0.961; Hepcidin, 0.976; STING1, 0.911; CYP2D6, 0.935; NK-βκ1, 1.113; TNF-α, 1.046; MUC2, 1.022; NK-βκ1 (liver), 0.998; TNF-α (liver), 1.100; AP, 1.015; SI, 1.032; Na^+^/K^+^ ATPase, 1.024.

### 2.5. Morphological Examination

The duodenum samples (*n* = 5 per treatment group) collected were fixed in fresh 4% (*v*/*v*) formaldehyde solution (stabilized with phosphate buffer), dehydrated, cleaned, and embedded in paraffin. Four sections (5 µm) of each repeat were taken of the duodenum and placed on a glass slide. Paraffin was removed using xylene, rehydrated using a series of graded alcohol, stained with Alcian Blue and examined under a light microscope (BX3M series, Olympus Waltham, MA, USA) using the CellSens Standard Software. The following variables were measured in each sample: villus height, villus width, goblet cell type, diameter and number (in epithelial villi and within crypts), number of Paneth cells per crypt and crypt depth. Ten villi per section (4 sections per biological repeat, 5 repeats per treatment group) were measured for statistical analysis.

### 2.6. Cecal Microbial DNA Isolation and Analysis

Weighed cecum samples (*n* = 5) (0.2 ± 0.02 g) were placed in the 2 mL PowerBead Tubes included in the PowerSoil^®^ DNA isolation kit and the protocol described by the manufacturer (MoBio Laboratories Ltd., Carlsbad, CA, USA) was followed. The extracted DNA was amplified for 16S rRNA gene sequences amplification from the V4 hypervariable region of microbial genomic DNA as previously described [37,41]. The samples were sequenced at Bar Ilan University (Safed, Israel) using an Illumina MiSeq Sequencer (Illumina, Inc., Madison, WI, USA). Amplicon reads were analyzed using “quantitative insights into microbial ecology” (QIIME2) and “Divisive Amplicon Denoising Algorithm” (DADA2) software [42,43]. A phylogenetic tree with taxonomic distribution was generated using Greengenes database. Shannon’s diversity index was used to assess α-diversity. The Bray–Curtis similarity index was used to assess β-diversity. The Bray–Curtis similarity index was used to assess β-diversity. When multiple groups were compared, q-values are presented following an FDR correction for multiple comparisons.

### 2.7. Duodenal Microbial DNA Isolation and Analysis

The duodenum was weighed and treated as described earlier [33,34,37,38,44]. Duodenum samples were weighed in sterile 15 mL tubes containing PBS. Sterile beads (3 mm in diameter) were added to the tubes and vortexed for 3 min. The pulverized duodenum was centrifuged at 700× *g* for 1 min, after which the supernatant was collected, and this was centrifuged again at 12,000× *g* for 5 min. The pellet so formed was washed with PBS twice and stored at −80 °C until DNA extraction. For the extraction, the pellet was treated with 50 mM EDTA and treated with lysozyme (Sigma Aldrich CO., St. Louis, MO, USA; final concentration of 10 mg/mL) for 45 min at 37 °C. Microbial DNA was isolated using a DNA purification kit (Promega, Madison, WI, USA). Primer for *Lactobacillus*, *Bifidobacterium*, *Lactiplantibacillus plantarum*, *Clostridium*, and *E. coli* were designed according to previously published data by Zhu et al. [45] and Pisula A. [46].

### 2.8. Statistical Analysis

The data in this paper are depicted as their mean values and standard error means. Experimental treatments and controls for intra-amniotic administration was assigned randomly after ensuring even weight distribution to all groups. ANOVA was used to analyze the results and *p*-values (*p* < 0.05) for significance were determined using post-hoc Duncan test. Software SPSS version 20.0 was used for statistical analysis.

## 3. Results

### 3.1. Body Weight, Cecum Weight and Blood Hemoglobin Concentration

There were no significant changes observed in the average hemoglobin concentration, average hatchling body or cecum weight among the different treatment groups and controls (*n* = 10).

### 3.2. Duodenal Gene Expression Analysis of Relevant Proteins

**Figure 1:**
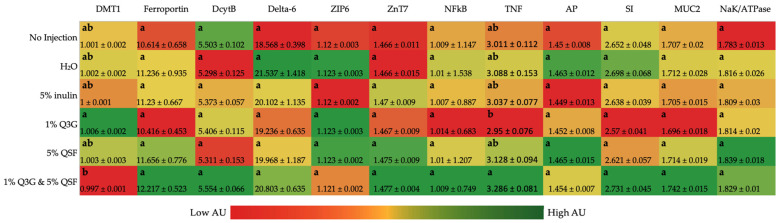
Effect of the intra-amniotic administration of treatments and controls on duodenal gene expression. Values are the means (AU: arbitrary units) ± SEM, *n* = 6. a,b genes (column wise) not indicated by the same letter are significantly different (*p* < 0.05). DMT1, Divalent metal transporter 1; Dcytb, duodenal cytochrome b; ZIP6, Zrt-, Irt-like proteins; ZnT7, zinc transporter 7; NF-κβ, nuclear factor kappa B; TNF-α, tumor necrosis factor; AP, Aminopeptidase; SI, Sucrose isomaltase; MUC2, mucin 2; Q3G, quercetin 3-glucoside; QSF, quinoa soluble fiber.

Figure 1 shows the gene expression of the different proteins associated with Zn and Fe metabolism, BBM functionality and immune/inflammatory responses. No significant differences were observed, except in the case of DMT1 and TNF-α. DMT1 is responsible for the transport of ferrous iron and other divalent mental ions out of the endosomal compartment and/or across the plasma membrane. DMT1 gene expression was down-regulated in the combination treatment group when compared to Q3G. On the contrary, TNF-α gene expression was up-regulated in the same group when compared to Q3G alone.

### 3.3. Liver Gene Expression Analysis of Relevant Proteins

**Figure 2:**
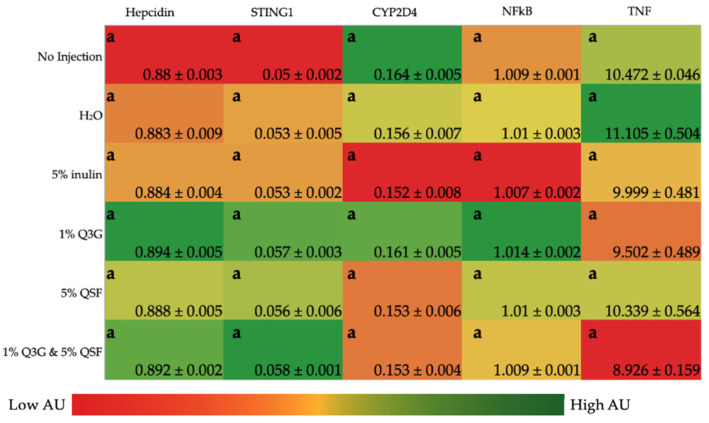
Effect of the intra-amniotic administration of treatments and controls on liver gene expression. Values are the means (AU: arbitrary units) ± SEM, *n* = 6. Values (column wise) are not statistically significant (*p* < 0.05). STING1, stimulator of interferon genes; Cyp2d4, cytochrome P450, family 2, subfamily d, polypeptide 4; NF-κβ, nuclear factor kappa beta; TNF-α, tumor necrosis factor. Q3G, quercetin 3-glucoside; QSF, quinoa soluble fiber.

As shown in Figure 2, the intra-amniotic administration of neither Q3G nor QFS resulted in significant differences in inflammation/immune response-related proteins (STING1, CYP2D4, TNF-α, NK-κβ) and hepcidin when compared to the controls in the *Gallus gallus* liver samples.

### 3.4. Duodenal Morphological Measurement

#### 3.4.1. Goblet Cell Number and Type (Villi and Crypt)

Table 2 depicts the morphometric measurements including goblet cell number and type in the duodenal epithelia villi and total goblet cell number in the crypts. In general, the treatments increased goblet cell number in both villi and crypts in comparison to the controls, with the greatest increase in villi goblet cell number seen in groups treated with QSF. The combination treatment of quinoa and quercetin resulted in the greatest crypt goblet cell number.

#### 3.4.2. Paneth Cell Number and Diameter

As seen in Table 3, the intra-amniotic administration of QSF, Q3G and their combination resulted in a significant (*p* < 0.05) increase in Paneth cell number relative to the controls. The 1% Q3G group resulted in the larger Paneth cell diameter (µM) and number.

#### 3.4.3. Average Villi Surface Area and Goblet Cell Diameter

The effect of intra-amniotic administration of treatments and controls on villi surface area and goblet cell diameter in duodenal villi and crypts are depicted in Table 4. Overall, the treatment groups and inulin resulted in a larger villi surface area and goblet cell diameter when compared to the control (no injection and H_2_O). QSF treatment resulted in the largest goblet cell diameter both in the crypts and villi. The inulin treatment resulted in the highest villi surface area.

### 3.5. Duodenal Microbial Populations

**Figure 3:**
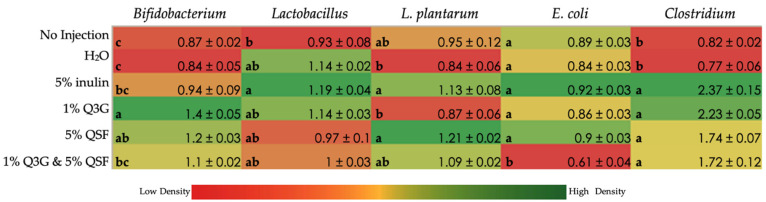
Effect of intra-amniotic administration of QSF and Q3G on genera and species level bacterial populations in the duodenum. Values are the means ± SEM, *n* = 6. Values (column wise) not indicated by the same letter are statistically significant (*p* < 0.05). Q3G, quercetin 3-glucoside; QSF, quinoa soluble fiber.

Figure 3 shows the relative abundance of probiotic *Bifidobacterium*, *Lactobacillus* and *Lactiplantibacillus plantarum*. Additionally, the relative abundance of *Clostridium* and *E. coli* (generally regarded as pathogenic bacteria) was measured. It was found that 5% inulin treatment, a known prebiotic, increased the populations of all bacterial groups assessed versus no injection control. A total of 5% QSF proved to be comparable to prebiotic inulin for its effects on the bacteria assessed. Interestingly, 1% Q3G and 5% QSF treatment groups showed a significant reduction in *E. coli* populations and a significant increase in *Clostridium* numbers compared to the controls.

### 3.6. Cecal Microbiota Analysis

**Figure 4:**
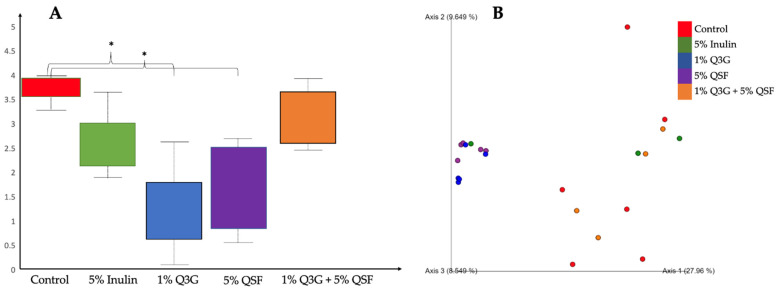
Cecum bacterial population analysis. (**A**) Measure of α-diversity using Shannon’s diversity index, the q value was established using Kruskal–Wallis (pairwise) test; (**B**) Principal Coordinate analysis (PCoA) showing β-diversity using the Bray–Curtis index, PERMANOVA (pairwise) test for statistical significance. n~5, * = *q* < 0.05. Q3G, quercetin 3-glucoside; QSF, quinoa soluble fiber.

Figure 4A shows the observed differences in the cecal microbiome between the different groups. α-diversity analysis using Shannon’s diversity index revealed significant differences between the control versus 1% Q3G and 5% QSF treatment groups. The latter two showed significant reduction in bacterial richness. Additionally, β-diversity analysis (Figure 4B) using the Bray–Curtis similarity index revealed clustering in the PCoA plot. The microbiomes of the animals that received 1% Q3G and 5% QSF were clustered together when compared to the other treatment groups. Groups 1% Q3G and 5% QSF were found to be statistically different (*q* < 0.05) from the control group (H_2_O + no injection).

**Figure 5:**
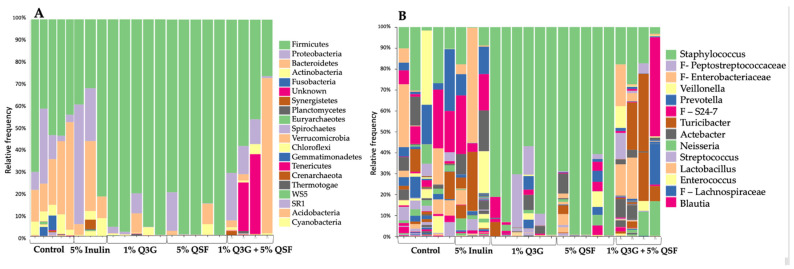
Compositional changes of the cecal microbiota in *Gallus gallus* following intra-amniotic administration. (**A**) Bacterial composition analysis at the phylum-level (**B**) Genus-level visualization of bacterial composition. F—family-level (when genus not known). Q3G, quercetin 3-glucoside; QSF, quinoa soluble fiber.

*Firmicutes* was the dominant phylum and *Staphylococcus* the dominant genus observed in the cecum of these treatment groups (Figure 5). The treatment group (1% Q3G + 5% QSF) visibly changed the cecal microbial composition when compared to the control. Additionally, 1% Q3G and 5% QSF seemed to favor the growth of *Staphylococcus* compared to other bacterial genera.

## 4. Discussion

In this study, we have demonstrated that the intra-amniotic administration of quinoa soluble fiber (QSF) in combination with quercetin-3-glucoside (Q3G) led to a significant (*p* < 0.05) improvement in brush border membrane (BBM) morphology and significant changes to the microbiome in vivo (*Gallus gallus*). The morphological data indicates that the chosen fiber together with the polyphenol exerted a prebiotic effect on the gut. This is the first study that looked at the synergistic potential of quinoa and quercetin on BBM morphology, functionality, and gut microbiome.

The results show that the intra-amniotic administration of the various treatment groups had no effect on blood hemoglobin concentration, cecum weight and overall body weight. This suggests that the chosen concentrations were not detrimental even to the naïve developing embryo. Several in vivo studies evaluating the effects of quinoa consumption have shown reduction in body weight [47,48], including a study on broilers [49], although the same has not been observed in clinical studies [3,50,51,52]. This may be because while preparing ready-to-eat quinoa for the clinical studies, the quinoa is thoroughly washed; this reduces the saponin content. Saponins are naturally occurring glycosides that have been shown to be an effective tool for body weight reduction [53]. In this study, as in the clinical trials, the quinoa seeds were purchased from a grocery store in ready-to-eat form; this is a likely explanation of the observed results with body weight. Alternatively, consumption for over a longer period of time is required to see changes in body weight.

The gene expression analysis (Figure 1) revealed no significant changes to the Fe transporter proteins (Ferroportin, DcytB), Zn transport proteins (ZIP6, ZnT7), Zn metabolism (Delta 6), proinflammatory protein (NF-κβ), nor BBM functionality proteins (AP, SI, MUC2, NaK/ATPase). This constancy suggests that prolonged consumption of Q3G and/or QSF is perhaps required to see changes in intestinal functionality. There were significant differences between 1% Q3G and the combination treatment (1% Q3G and 5% QSF) in DMT1 and TNF expression. Previous studies have linked inflammation to reduced mineral absorption [54], although the expression of the two were not significantly different when compared to the control groups (no injection, H_2_O). Despite a reduction in DMT1 expression and increase in TNF expression, there were no changes observed in hemoglobin concentrations and liver inflammatory proteins (STING1, Cyp2d4, NF-κβ, TNF) (Figure 2) suggesting that the combination treatment does not have any overall negative effects, as seen in this study.

Our results (Table 2, Table 3 and Table 4) show that the combination treatment beneficially altered the BBM morphology. The intra-amniotic administration of 1% Q3G and 5% QSF resulted in significantly greater numbers of crypt and acidic villi goblet cells when compared to the controls and 5% inulin. Goblet cells are specialized secretory cells that line the epithelial layer with mucin glycoproteins. These proteins protect the BBM and harbor the gut microbiome. A larger number of goblet cells is indictive of greater mucin production capacity [55]. Acidic goblet cells have the potential to lower luminal pH which in turn affects the resident bacteria. In Table 2, we see a significant difference between the goblet cell types (mixed, acidic, and neutral) corresponding to the different treatments. We hypothesize that the fiber in quinoa, through certain cellular signal(s), led to the differentiation of secretory progenitor cells [55] into acidic goblet cells. The polyphenol (Q3G) induced a different cell signal leading to the formation of more mixed goblet cells than acidic. This is the first demonstration of the effect of dietary fiber on acidic goblet cell proliferation. Further investigation is needed to characterize these pathways.

Table 3 shows that Paneth cell number and diameter was significantly higher in the group administered with 1% Q3G compared to the others. Paneth cells secrete antimicrobial peptides that are essential for defense against intestinal pathogens [56]. As with the goblet cells, a higher number and diameter indicate greater ability of Paneth cells to secrete these antimicrobial peptides, therefore suggesting better immune defense. It is important to note that the combination treatment group did significantly better on these parameters compared to 5% inulin, a well-established prebiotic fiber known to beneficially modulate the BBM [57,58,59,60]. Inulin, a naturally occurring polysaccharide, has been shown to increase villi surface area, i.e., stimulate cellular proliferation increasing the absorptive surface previously [59]. The same is observed in here. Table 4 shows that 5% inulin corresponded to the largest surface area followed by 1% Q3G and the combination treatment group.

Much as with the human microbiome, *Gallus gallus* too harbors an active and complex microbiome that is susceptible to changes in diet [31,45]. The bacterial populations were assessed both in the duodenum (the cite of mineral absorption) and cecum (region where majority of the bacteria reside). A 2018 study by Zmora et al. has shown that bacterial populations vary throughout the gastrointestinal tract [61]. This is consistent with our findings wherein the microbiome differed in the duodenum and cecum in *Gallus gallus* within individual organisms. The duodenal 16s rDNA analysis (Figure 3) showed that 1% Q3G increased the abundance of health-promoting bacteria *Bifidobacterium* (*p* < 0.05) and *Lactobacillus* relative to the controls. This is in line with previous studies that have shown that polyphenols promote the growth of these two bacterial populations [62]. *L. plantarum* is a lactic acid-fermenting bacteria that reduces the luminal pH, activates phytases and increases mineral absorption [63]. A total of 5% QSF showed the highest abundance of *L. plantarum* relative to the other groups. The 5% QSF also corresponds to the highest number of goblet cells in the villi. We hypothesize that the increase in mucin proteins (due to increased number of goblet cells) in the duodenum has led to the growth of *L. plantarum* [64]. Alternatively, most strains of *E. coli* are regarded as pathogenic that cause intestinal infections. The combination treatment caused a significant (*p* < 0.05) reduction in *E. coli* numbers relative to the controls. On the contrary, the *Clostridium* numbers increased in all treatment groups relative to the controls. A previous study, assessing the effects of pseudo cereals quinoa and amaranth in vitro, demonstrated an increase in two *Clostridium* sp. [65]. This observation suggests that the growth of certain bacteria within genus *Clostridium* is supported by dietary quinoa. Quinoa may be metabolized by bacteria within the *Clostridium* genus.

The taxon-based 16s rRNA gene sequencing revealed that most of the bacterial sequences were dominated by Firmicutes, followed by Proteobacteria and Bacteroidetes as seen in Figure 5A. At the genus level, it is seen that *Staphylococcus* sp. dominated the sequences, especially in the 1% Q3G and 5% QSF groups (Figure 5B). *Staphylococci* are readily found in the epithelial surface of the human gut; their role, however, is not well established. Some species of this genus are considered to have no pathogenicity to their host, while others can be infectious [66]. Further analysis is required to establish causation for this correlation observed in quercetin and quinoa consumption to increase in Staphylococci numbers. The changes observed here are consistent with our α-diversity and β-diversity analysis. The intra-amniotic administration of 1% Q3G and 5% QSF significantly (*p* < 0.05) reduced α-diversity relative to the control group (combination of no injection and H_2_O). The two groups also showed clustering in the PCoA plot created using the Bray–Curtis index. The combination treatment (1% Q3G + 5% QSF), however, did not result in reduced bacterial diversity, but was found to be different relative to the controls. These results indicate that the combination treatment modulated the gut microbiome without reducing microbial diversity.

The strength and limitations of this study both lie in its methodology. This intra-amniotic in vivo technique uses the embryonic phase of *Gallus gallus*. It is a well-established technique to assess bioactive compounds in vivo [30,31,32,33,34,35,36,37,38,39,40,67]. The egg is a complete and isolated system, ensuring that the given intra-amniotic treatment is the only variable. Therefore, the results at the molecular, physiological, and morphological level are due to the given intervention alone. On the other hand, this is a preliminary study with only a short-term exposure to the test substances. Further long-term studies would be required to assess the effects of repeated consumption along with a complete diet.

## 5. Conclusions

The data presented in this study suggests that the consumption of quercetin-3-glucoside and quinoa soluble fiber can beneficially affect the duodenal brush border membrane morphology and duodenal microbial populations. Studies including prolonged consumption of the two are required to further establish synergy. This is the first study to demonstrate the potential synergistic effect of the chosen soluble fiber and polyphenol as a prebiotic.

## Figures and Tables

**Table 1:** DNA sequences of primers used in this study.

Analyte	Forward Primer (5′→3′)	Reverse Primer (5′→3′)	Base Pair	GI Identifier
Iron Metabolism				
DcytB	CATGTGCATTCTCTTCCAAAGTC	CTCCTTGGTGACCGCATTAT	103	20380692
DMT1	TTGATTCAGAGCCTCCCATTAG	GCGAGGAGTAGGCTTGTATTT	101	206597489
Ferroportin	CTCAGCAATCACTGGCATCA	ACTGGGCAACTCCAGAAATAAG	98	61098365
Hepcidin *	AGACGACAATGCAGACTAACC	CTGCAGCAATCCCACATTTC	132	SAMN08056490
CYP2D6 *	GATTCCTGCCTCAGCTTCTT	CCAGGTCTCCTTGTGCTTATC	134	417981
Zinc Metabolism				
Δ-6-desaturase	GGCGAAAGTCAGCCTATTGA	AGGTGGGAAGATGAGGAAGA	93	261865208
ZIP6	GCTACTGGGTAATGGTGAAGAA	GCTGTGCCAGAACTGTAGAA	99	66735072
ZnT7	GGAAGATGTCAGGATGGTTCA	CGAAGGACAAATTGAGGCAAAG	87	56555152
Inflammatory Response				
NF-κβ *	CACAGCTGGAGGGAAGTAAAT	TTGAGTAAGGAAGTGAGGTTGAG	100	2130627
TNF-α *	GACAGCCTATGCCAACAAGTA	TTACAGGAAGGGCAACTCATC	109	53854909
STING1 *	CTCCTTGTGAAGGTCTTCTCTG	GGACGTCTCCTTATGTTGATGG	99	768990
BBM functionality				
AP	CGTCAGCCAGTTTGACTATGTA	CTCTCAAAGAAGCTGAGGATGG	138	45382360
SI	CCAGCAATGCCAGCATATTG	CGGTTTCTCCTTACCACTTCTT	95	2246388
MUC2	CCTGCTGCAAGGAAGTAGAA	GGAAGATCAGAGTGGTGCATAG	155	423101
Na^+^/K^+^ ATPase	CCTTGGAGGTTTCTTCACCTATT	GGTCATCCCACTGAAGTCTAATC	92	14330321

Dcytb, duodenal cytochrome b; DMT1, Divalent metal transporter 1; Cyp2d4, cytochrome P450, family 2, subfamily d, polypeptide 4; ZIP6, Zrt-, Irt-like proteins; ZnT7, zinc transporter 7; NF-κβ, nuclear factor kappa beta; TNF-α, tumor necrosis factor; STING1, stimulator of interferon genes; SI, Sucrose isomaltase; AP, Aminopeptidase; MUC2, mucin 2; GI, gene identifier. * Analyzed in the liver.

**Table 2:** Effect of intra-amniotic administration of treatments and controls on goblet cell type and total number of goblet cells in duodenal villi and crypts.

Treatment Group	Average Goblet Cell Number in the Villi			Total Villi Goblet Number	Total Crypt Goblet Number
	Acidic	Neutral	Mixture		
No Injection	13.59 ± 0.39 ^d^	0.01 ± 0.01 ^c^	3.50 ± 0.23 ^cd^	17.09 ± 0.49 ^d^	6.95 ± 0.21 ^d^
H_2_O	15.03 ± 0.39 ^cd^	0.01 ± 0.01 ^c^	5.76 ± 0.30 ^b^	20.80 ± 0.47 ^c^	7.83 ± 0.19 ^c^
5% Inulin	16.39 ± 0.54 ^bc^	0.10 ± 0.02 ^bc^	6.53 ± 0.30 ^ab^	23.02 ± 0.60 ^b^	9.15 ± 0.16 ^b^
1% Q3G	17.58 ± 0.66 ^b^	0.32 ± 0.07 ^a^	7.22 ± 0.40 ^a^	25.11 ± 0.75 ^a^	9.33 ± 0.17 ^b^
5% QSF	21.68 ± 0.79 ^a^	0.21 ± 0.05 ^ab^	4.58 ± 0.37 ^c^	26.46 ± 0.89 ^a^	9.38 ± 0.17 ^b^
1% Q3G and 5% QSF	22.38 ± 0.67 ^a^	0.06 ± 0.02 ^c^	3.21 ± 0.21 ^d^	25.65 ± 0.69 ^a^	9.98 ± 0.18 ^a^

Values are the means ± SEM, *n* = 5. ^a–d^ Treatment groups not indicated by the same letter are significantly different (*p* < 0.05). Q3G, quercetin 3-glucoside; QSF, quinoa soluble fiber.

**Table 3:** Effect of intra-amniotic administration of treatments and controls on Paneth cell number and diameter.

Treatment Group	Paneth Cell Number	Paneth Cell Diameter (µM)
No Injection	1.48 ± 0.05 ^d^	1.67 ± 0.03 ^c^
H_2_O	2.46 ± 0.11 ^c^	1.82 ± 0.04 ^b^
5% Inulin	2.56 ± 0.09 ^c^	1.68 ± 0.03 ^c^
1% Q3G	3.61 ± 0.14 ^a^	1.94 ± 0.04 ^a^
5% QSF	3.26 ± 0.13 ^b^	1.47 ± 0.03 ^d^
1% Q3G and 5% QSF	3.13 ± 0.11 ^b^	1.86 ± 0.04 ^ab^

Values are the means ± SEM, *n* = 5. ^a–d^ Treatment groups not indicated by the same letter are significantly different (*p* < 0.05). Q3G, quercetin 3-glucoside; QSF, quinoa soluble fiber.

**Table 4:** Effect of intra-amniotic administration of treatments and controls on villi surface area and goblet cell diameter in duodenal villi and crypts.

Treatment Group	Average Surface Area (mm^2^)	Villi Goblet Cell Diameter (µM)	Crypt Goblet Cell Diameter (µM)
No Injection	112.51 ± 4.28 ^e^	2.39 ± 0.04 ^d^	2.68 ± 0.02 ^b^
H_2_O	143.33 ± 5.27 ^de^	3.11 ± 0.03 ^b^	2.65 ± 0.02 ^b^
5% Inulin	206.92 ± 6.37 ^a^	2.74 ± 0.03 ^e^	2.51 ± 0.02 ^c^
1% Q3G	173.60 ± 4.66 ^b^	2.94 ± 0.03 ^c^	2.47 ± 0.02 ^c^
5% QSF	156.50 ± 4.85 ^cd^	3.58 ± 0.03 ^a^	2.98 ± 0.02 ^a^
1% Q3G and 5% QSF	162.83 ± 5.24 ^bc^	3.02 ± 0.03 ^c^	2.50 ± 0.02 ^c^

Values are the means ± SEM, *n* = 5. ^a–e^ Treatment groups not indicated by the same letter are significantly different (*p* < 0.05). Q3G, quercetin 3-glucoside; QSF, quinoa soluble fiber.

## Data Availability

Not applicable.
